# Degradation of Silicone Rubbers as Sealing Materials for Proton Exchange Membrane Fuel Cells under Temperature Cycling

**DOI:** 10.3390/polym10050522

**Published:** 2018-05-13

**Authors:** Fan Wu, Ben Chen, Yizhi Yan, Yanan Chen, Mu Pan

**Affiliations:** 1 State Key Laboratory of Advanced Technology for Materials Synthesis and Processing, Wuhan University of Technology, Wuhan 430070, China; wu_fan@whut.edu.cn (F.W.); yanyizhi2005@163.com (Y.Y.); anyc8712@whut.edu.cn (Y.C.); 2 Hubei Key Laboratory of Fuel Cells, Wuhan University of Technology, Wuhan 430070, China; 3 Hubei Key Laboratory of Advanced Technology for Automotive Components, Wuhan University of Technology, Wuhan 430070, China

**Keywords:** silicone rubbers, seals, degradation, temperature cycling, PEMFC

## Abstract

Gaskets are compressed in proton exchange membrane fuel cells (PEMFCs) to keep fuel, oxidant and coolant within their respective regions and are very important for sealing and maintaining electrochemical performance of fuel cells during their long-term operation. It has been proved that the gas leakage caused by the failure of the gaskets following long-term operation is one of the main reasons for PEMFC performance degradation. In this work, degradation of silicone rubbers, the potential gasket materials for PEMFCs, were investigated in the simulated PEMFC environment solution, weak acid solution, de-ionized water and air, respectively, under alternating temperature cycling from −20 °C to 90 °C. The changes in hardness, weight, chemical properties, mechanical behavior and surface morphology of the samples of silicone rubbers were studied after a certain number of temperature cycles. The results show that with the increase in temperature cycles, the hardness of the samples increases and the weight of the samples decreases gradually. Scanning electron microscopy reveals that cracks and caves constantly appear on the surface of the samples. Attenuated total reflection Fourier transform infrared spectra (ATR-FTIR) results demonstrate that the surface chemistry changes via de-crosslinking and chain scission in the backbone due to the exposure of samples to the environments over time under alternating temperature cycles.

## 1. Introduction

Fuel cells are energy conversion devices which transfer the chemical energy of hydrogen and oxygen into electricity directly, and are regarded as renewable, promising and environment friendly energy sources [1,2]. Of different types of fuel cells developed, proton exchange membrane fuel cell (PEMFC) is considered to be the most promising and attractive candidate in portable and transportation applications due to the additional advantages of high energy density, fast start-ups and low operating temperature and it thus becomes the focus of research currently [3,4]. However, the durability and reliability of PEMFC are remain significant challenges for its commercial applications [5,6,7].

Gaskets and seals are essential parts for the durability and stability of the PEMFC stack, which are applied to keep the reactant gases and coolant within their respective regions. During the operation of PEMFCS, leakage cannot be neglected, as it not only affects the electrochemical performance of the cell, but also reduces its lifetime. Even worse, it may cause unexpected accidents owing to the mixing of hydrogen and oxygen [8]. Sealing materials should possess the ability of excellent stability and reliability under long-term operation of PEMFC [6,8,9]. Elastomeric seals in PEMFC are exposed to the surroundings of hydrogen, humidified air and acidic environment. Moreover, they suffer from mechanical stress as well as elevated temperature [10,11,12,13,14]. The long-term stability and durability of the elastomeric seal materials are crucial to both sealing and electrochemical performance of PEMFC. Thus, researchers have paid more and more attention to the degradation of the elastomeric gasket material for PEMFC.

Silicone is considered to be a potential candidate for PEMFC due to low cost, good mechanical properties and easy manufacturing process [15,16]. There are many reports focusing on the chemical and mechanical stability of silicone rubbers in various testing environments [17,18,19,20,21,22]. Li et al. [16,17] investigated the degradation of silicone rubbers exposed to simulated and accelerated proton exchange membrane fuel cell environments. They found that the concentration of the acid in test solutions has a significant effect on the degradation of silicone rubbers. Feng et al. [23] investigated degradation of silicone rubbers with different hardness in various aqueous solutions. They found that silicone rubbers became more durable in aqueous solutions with the increase in hardness. Schulze et al. [24] reported the degradation of seals during PEMFCs operation. They found that the small particles released from silicone rubbers could migrate to the membrane electrode assembly and lower the electrochemical performance of the fuel cell. Tan et al. [25,26,27,28,29] studied the degradation of silicone rubbers exposed to PEMFC environment. The result showed that silicone rubbers degraded more severely with the increasing acid and temperature, the degradation of silicone rubbers was attributed to chemical decomposition of silicon-based backbone accompanied with leaching of fillers. Cui et al. [30,31] studied the sealing force and thermal stress development of silicone rubbers under temperature cycling. They found that thermal expansion or contraction was the major contributor to the compressive stress developed in the silicone rubbers seal.

It is restricted by proton conduction and current formation mechanisms that operating temperature of PEMFCs is often below 80 °C [32]. In order to improve heat management, the increase in operating temperature is the trend of PEMFCs [33,34,35,36]. Based on the state of the art of fuel cell technology, many fuel cell companies including General Motors have increased the operating temperature up to 90 °C [37,38]. For the cold start of fuel cell, most research has mainly focused on the cold start strategies and lower cold start temperature, whereas the effect of the low temperature environment on the material was ignored [39]. The aging mechanism of sealing materials in low temperature environment below zero is still blank. Although various simulated or accelerated PEMFCs environments have been selected to study the degradation of silicone rubbers, little work involving with the degradation of silicone rubbers under alternating temperature cycling has been carried out, especially the high working temperature up to 90 °C demanding for fuel cell development and low temperature below to −20 °C for cold start.

In this study, the degradation of silicone rubbers exposed to different environments under alternating temperature cycling from −20 °C to 90 °C was investigated in detail. The aim is to investigate the degree of degradation and degradation mechanisms for the silicone rubber samples exposed to different testing environment over time under alternating temperature cycling condition. The regular solution [24,26] similar to the actual PEMFCs environment and three other environments, including weak acid solution, deionized water and air were prepared. The weight and hardness changes of the silicone rubber samples were monitored at every certain number of temperature cycles. In addition, scanning electron microscopy (SEM) of the surface of the samples was compared. Besides, the chemistry changes of the silicone rubber were analyzed by attenuated total reflection Fourier transform infrared spectra (ATR-FTIR) spectroscopy after every temperature cycle. The purpose of this study is to establish a mechanism for the deterioration of the sealing material, so as to predict the fuel cell sealing performance. The result will provide theoretical basis for the long life requirement of commercial fuel cells.

## 2. Experimental

### 2.1. Material and Test Environments

**Figure 1:**
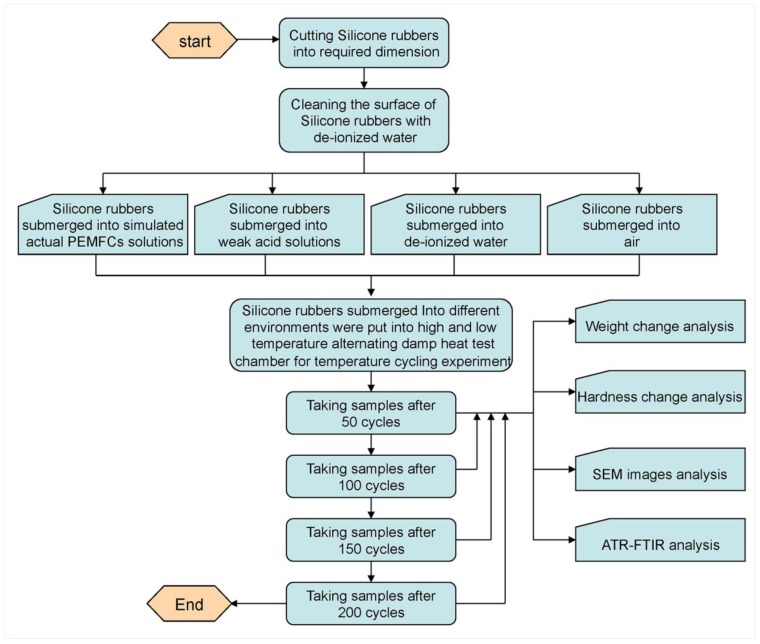
Testing process of silicone rubbers.

Methylvinyl silicone rubbers (Silicone Rubber Products Co., Ltd., Dongguan, China) with the hardness and thickness of 50 (Shore A) and 0.5 mm, respectively, were the investigated objects in this study. All the tested samples were rectangular with dimensions of 10.0 cm in length and 4.0 cm in width (seen in Table 1). Water used for solution preparation was de-ionized through a Milli-Q system (Barnsted Nanopore, resistivity = 18.0 MΩ cm^−1^, Chengdu Super Pure Technology Co., Ltd., Chengdu, China). Three solutions with different pH values and air were used as testing environment to study the degradation of silicone rubbers. Reagent grade acetic acid, sulfuric acid (98%), and hydrofluoric acid (48%) were brought from Sinopharm Chemical Reagent Co., Ltd. (Beijing, China). The first solution with a pH value of 3.35 is simulating actual PEMFCs working environment, with chemical composition of 12 ppm H_2_SO_4_ and 1.8 ppm HF with reagent grade. The second testing environment is acetic acid solution with pH value of 5.0. The third liquid environment with a pH value of 7.0 was de-ionized water. All the samples exposed to different testing environment were placed in high and low temperature alternating damp heat test chamber (Wuxi Zhuo Cheng Test Equipment Co., Ltd., Wuxi, China) for alternating temperature cycling test (Figure 1). The temperature range of the test chamber is −40 °C~+150 °C. The heating rate for the alternation is from 1 °C/min to 2.5 °C/min, while the cooling rate is from 0.3 °C/min to 1 °C/min.

### 2.2. Aging and Characterization Methods

Prior to the temperature cycling test, four groups of silicone rubber samples were prepared, and were flushed with de-ionized water for cleaning the surface. Then the samples were submerged into three solutions and air, respectively, and placed in the high and low temperature alternating damp heat test chamber for degradation test. Since the typical PEMFC operating temperature is ranging from 65 °C to 90 °C, and cold start temperature is generally −20 °C, the temperature range of silicone rubbers degradation tests was conducted from −20 °C to 90 °C, which is considered to be a temperature cycle with 6 h. The degraded samples were taken out after every 50 temperature cycles, and then were thoroughly rinsed with de-ionized water and dried in air for further analysis. All the samples were conducted with 200 temperature cycles in this study.

Microelectronic balance (Boyue Instrusment Co., Ltd., Shanghai, China) was used to monitor the changes in weight of the samples. Before and after every 50 temperature cycles of silicone rubbers degradation test the weight of each simple was recorded for comparison. Before measuring the weight, the samples were carefully cleaned using reagent-grade water with 18 MΩ cm^−1^ resistances, followed by air-dried at room temperature for two hours. The hardness of cylindrical disc samples was measured by a Shore A durometer (Beijing Wowei Technology Co., Ltd., Beijing, China) following the ASTM D 2240 standard. The applied test pressure was just sufficient to ensure close contact of the durometer with the test sample, while recording the hardness immediately after close contact has been established. The test samples were shifted to the new position after each contact in order to avoid errors due to fatigue effects. For multilayered samples, the compression force was applied to the hard layer side to sustain the main compression force in PEMFC. A minimum of five readings were taken to give the average value. Attenuated total reflection Fourier transform infrared spectra (Thermo Nicolet Corporation, Madison, WI, USA) were recorded with a resolution of 4 cm^−1^ to investigate the chemical degradation of silicone rubbers. The surface morphology of silicone rubbers were analyzed by scanning electron microscopy (JEOL, Tokyo, Japan).

## 3. Results and Discussion

### 3.1. Weight Change

The silicone rubber samples were taken out from the test chamber after every 50 temperature cycles. The percentage of weight change was calculated using the following equation:

(1) $$W\left(\%\right)=\frac{W_{n}-W_{0}}{W_{0}}\times 100\%$$

where, *W*_0_ and *W*_n_ are the weight of the sample before temperature cycling test and after n cycles of alternating temperature cycling test, respectively.

**Figure 2:**
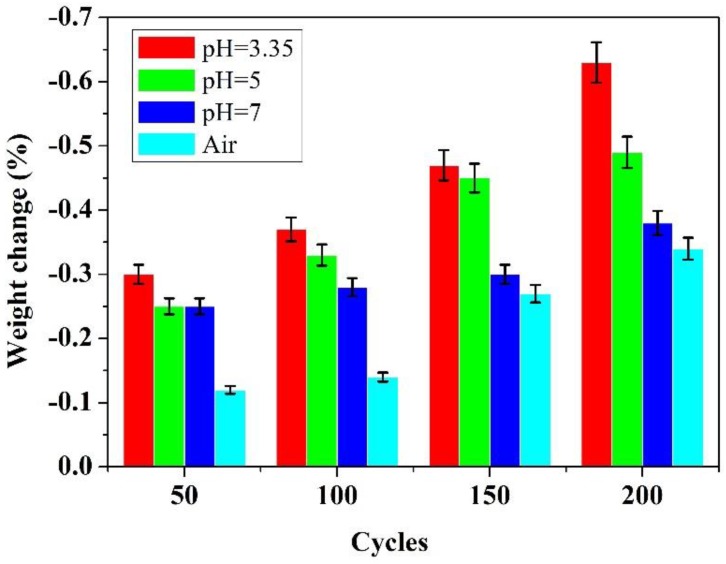
Weight change under temperature cycling.

Figure 2 shows the weight change of samples exposed to different testing environment under temperature cycling from −20 °C~90 °C, it is the accumulated weight loss since the very beginning. It is evident that the weight loss was observed for all samples after 200 cycles. The weight of the samples exposed to the solutions decreased apparently, especially exposed to the acidic solution with a pH value of 3.35. While the samples exposed to air did not change as much as those exposed to the solutions. It is apparent that the weight loss of the tested material increased with the increase in acidity of the exposed solution, the weight loss reached 0.63% in simulated acid solution with a pH value of 3.35 after 200 cycles, whereas it was 0.34% when exposed to air. Typically, fillers including silicon dioxide and calcium carbonate are often impregnated into silicone rubbers to improve their mechanical properties. When exposed to aqueous solution, the polysiloxane backbone of silicon rubbers swelled up and caused the fillers to leach out, leading to the weight loss of the samples [23]. Some of the fillers could react with acid in the solutions. In addition, the weight loss under these conditions was also due to the release of cyclic siloxanes [12]. The existence of F^−^ in the simulated solutions could also result in the chemical decomposition of silicone rubbers under acidic environment [23]. Thus, the weight loss in the simulated solution is more pronounced. Those results show that alternating temperature cycles and testing environment had great effect on the weight change of the samples. The weight loss increases with the increase in cycle numbers.

Table 2 shows the weight change of the samples between every 50 cycles. It indicated that the weight loss of the samples exposed to solutions is more serious than that exposed to air. The weight loss rate also increased with the increase in the acidity of the solution. While it was gradually reduced with the increase in cycle numbers, which indicate that fillers leach out rate became slow during the experiment.

### 3.2. Hardness Change

**Figure 3:**
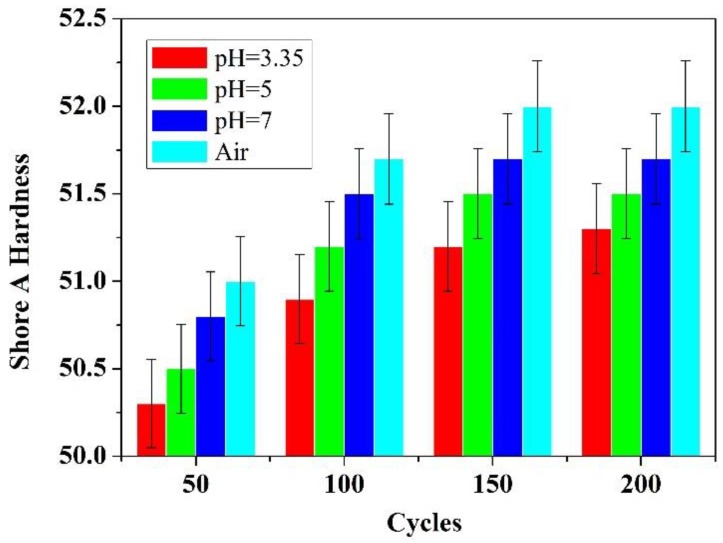
Shore A hardness change under temperature cycling.

The Shore A hardness of the samples was measured by a Shore A durometer. The hardness change is shown in Figure 3. The results show that the Shore A hardness of the samples was slightly increased after alternating temperature cycling test, which implying that the samples became harder on their surface layers. The Shore A hardness of the samples exposed to solutions increased more slowly than those exposed to air. The Shore A hardness of the samples increased to 52 when exposed to air while it increased to 51.25 when exposed to the acidic solution with a pH value of 3.35 after 200 cycles.

The hardness of the samples surface was increased due to the polymer chains could not explore to different configurations and behavior as less flexible, with the result that the material became more cross-linked [40,41,42]. Fillers impregnated into silicone rubbers could enhance their hardness, and it could leach out during the alternating temperature cycling test. Although fillers leached from silicone rubbers could reduce the hardness of samples [43], the further crosslink of silicone rubbers leaded to the hardness of the samples surface dominated. Therefore, the samples exposed to air were harder under the same cycling temperature with less weight loss.

### 3.3. Scanning Electron Microscopy

**Figure 4:**
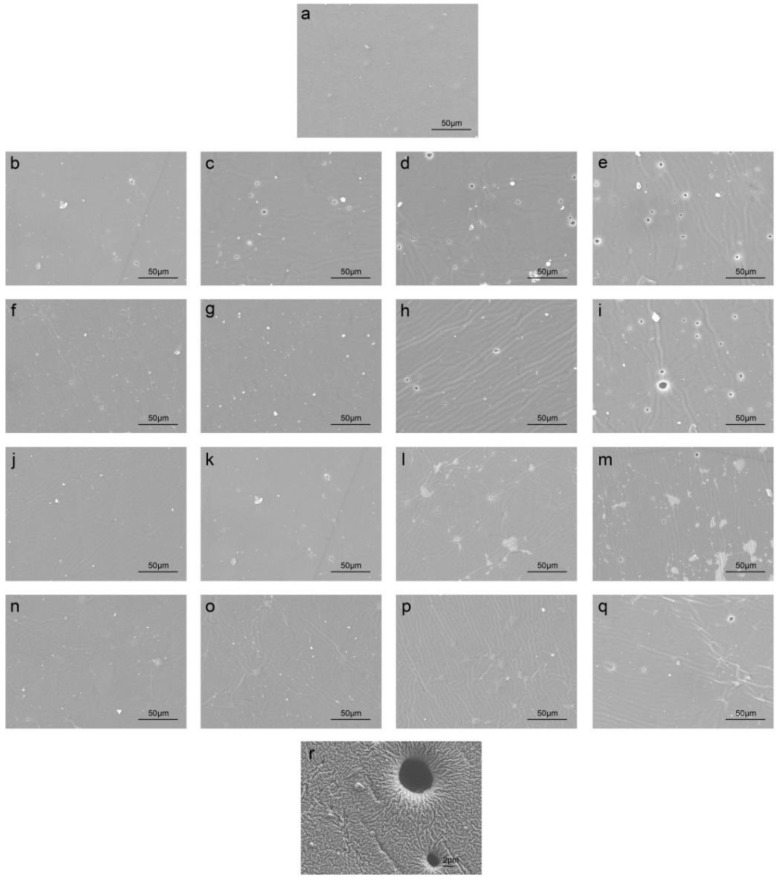
SEM images of silicone rubbers exposed to the environments under temperature cycling from −20 °C~90 °C, (**a**) original; (**b**) 50 cycles in pH = 3.35 solution; (**c**) 100 cycles in pH = 3.35 solution; (**d**) 150 cycles in pH = 3.35 solution; (**e**) 200 cycles in pH = 3.35 solution; (**f**) 50 cycles in pH = 5 solution; (**g**) 100 cycles in pH = 5 solution; (**h**) 150 cycles in pH = 5 solution; (**i**) 200 cycles in pH = 5 solution; (**j**) 50 cycles in de-ionized water; (**k**) 100 cycles in de-ionized water; (**l**) 150 cycles in de-ionized water; (**m**) 200 cycles in de-ionized water; (**n**) 50 cycles in air; (**o**) 100 cycles in air; (**p**) 150 cycles in air; (**q**) 200 cycles in air; (**r**) voids and cracks.

Figure 4 displays the SEM images of silicone rubbers before and after being exposed to the testing environment with different cycles. The magnification of Figure 4a–q was 500×, and the magnification used in Figure 4r was 2000×. The initial sample (Figure 4a) has rather smooth surface and no voids or cracks were observed. Figure 4b–e show the surface topographical changes of the silicone rubber before and after exposure to the simulated solution for 50 cycles, 100 cycles, 150 cycles and 200 cycles at the alternating temperature from −20 °C~90 °C, respectively. It can be seen that voids or cracks were formed on the surface of the sample after 50 cycles. In addition, these voids or cracks continuously grew with the increase of cycles.

Figure 4f–i show the surface topographical changes of the silicone rubber after exposure to the acetic acid solution for 50 cycles, 100 cycles, 150 cycles and 200 cycles at the alternating temperature from −20 °C~90 °C. It can be seen that cracks occurred on the surface of the sample after 50 cycles. Then cracks or voids grew with the increase of alternating temperature cycles.

Figure 4j–m show the surface topographical changes of the silicone rubber after exposure to de-ionized water for 50 cycles, 100 cycles, 150 cycles and 200 cycles at the alternating temperature from −20 °C~90 °C. After 50 cycles the surface condition of the samples exposed to the testing environment did not change significantly. Cracks were gradually observed and the surface almost kept smooth. It was until 100 cycles that there were voids or cracks formed on the surface of the samples exposed to the environments.

Figure 4n–q show the surface topographical changes of the silicone rubber after exposure to air for 50 cycles, 100 cycles, 150 cycles and 200 cycles at the alternating temperature from −20 °C~90 °C. It can be seen that the samples changed a little and no voids or cracks were observed before 200 cycles.

Figure 4r shows the magnified voids and cracks formed on the surface of the samples exposed to the simulated solution after 200 cycles. It can be seen that obvious voids and cracks appeared on the surface of the sample, thus leading to a decline in sealing performance.

From the scanning electron microscopy results, it can be concluded that the surface damage of the silicone rubber samples in the solutions is more serious than that in air. The increase in acidity and the temperature cycle numbers also results in significant damage of silicone rubbers [17]. The SEM result agrees with the above discussed variation of weight and hardness of silicone rubbers exposed to the environments under temperature cycling.

### 3.4. ATR-FTIR

**Figure 5:**
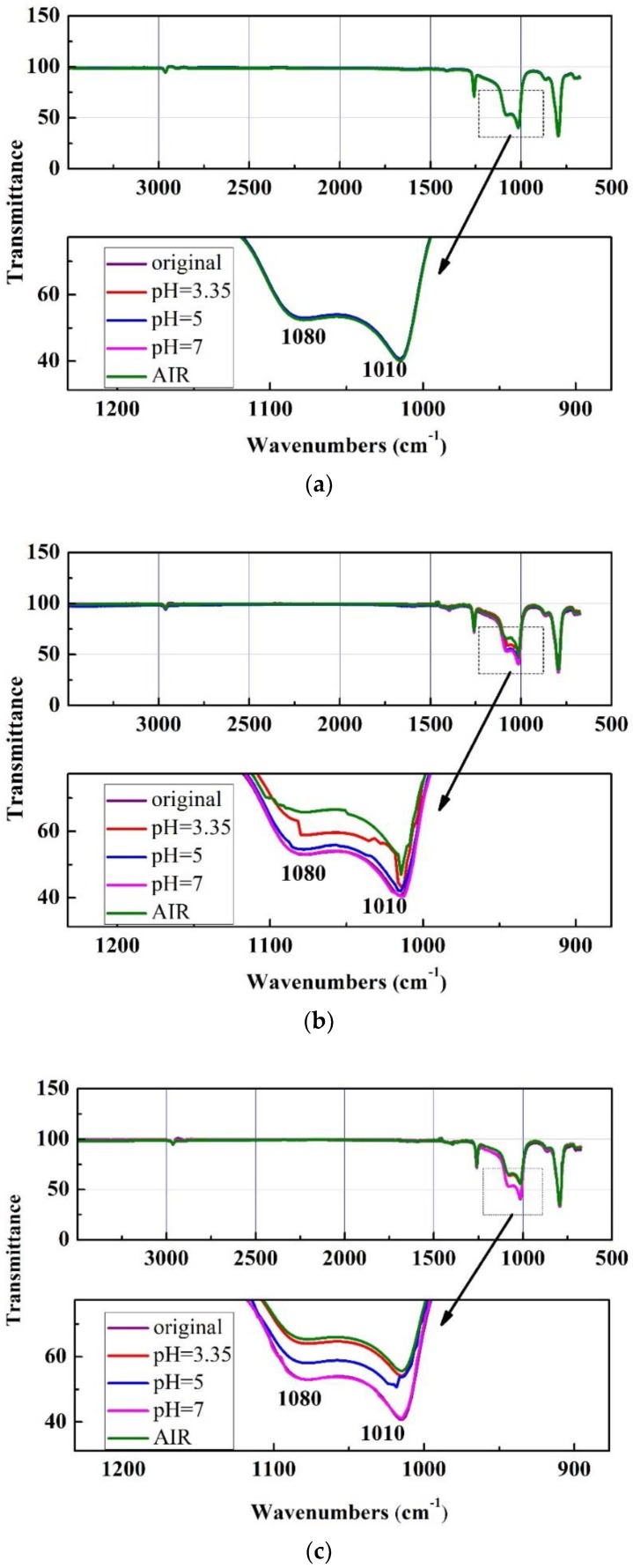

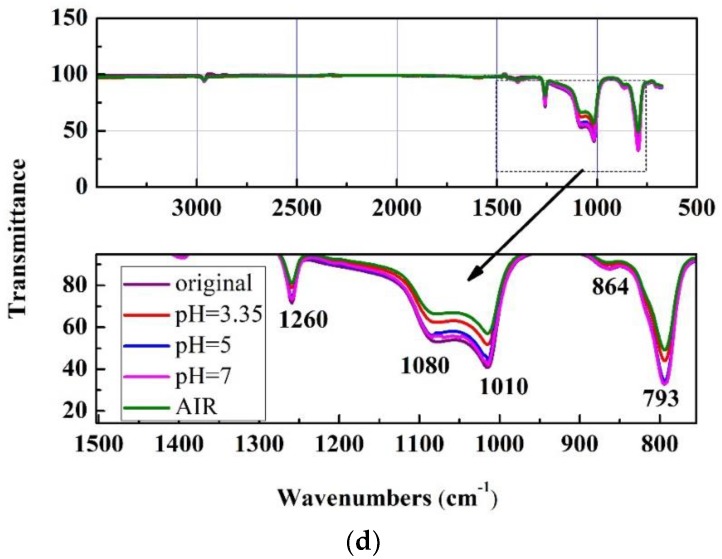
ATR-FTIR spectra of silicone rubbers exposed to the environments under temperature cycling from −20 °C~90 °C after different cycles, (**a**) 50 temperature cycles; (**b**) 100 temperature cycles; (**c**) 150 temperature cycles; (**d**) 200 temperature cycles.

ATR-FTIR spectra of silicone rubbers after exposed to the different environments under temperature cycling with 200 cycles were recorded in Figure 5. Figure 5a–d represent the ATR-FTIR results of the samples exposed to the simulated PEM fuel cell solutions, acetic acid solutions, de-ionized water and air under temperature cycling from 50 to 200 cycles, respectively. After being exposed to the environments under temperature cycling for certain times, samples were taken into ATR-FTIR spectra to determine the chemistry changes as a chemical degradation. ATR is an IR sampling technique for surface analysis. Place the testing surface of the sample directly on the germanium crystal, fix the sample, and collect the attenuated total reflection infrared spectrum by ATR-FTIR spectra. The strongest and broadest absorption bands for the samples are between 1010 cm^−1^ and 1080 cm^−1^, and there are other absorption bands at 793 cm^−1^, 1260 cm^−1^, 864 cm^−1^ and 2962 cm^−1^. In general, the absorption band at 2960 cm^−1^ is assigned to stretching vibration of –CH_3_. The absorption bands at 1260 cm^−1^ and 864 cm^−1^ are assigned to bending vibration and rocking vibration of Si–CH_3_. The absorption bands at 1080 cm^−1^ and 1010 cm^−1^ are assigned to the stretching vibration of Si–O–Si bonds on the backbone of silicone rubbers. The absorption band at 793 cm^−1^ is assigned to the coupling of stretching vibration of Si–C and rocking vibration of –CH_3_.

For silicone rubber samples exposed to the testing environments under the alternating temperature cycling from −20 °C to 90 °C up to 50 cycles (Figure 5a), the intensity of absorption bands had no obvious changes. This result reveals that the surface chemistry on the surfaces of silicone rubber samples did not change apparently in the testing environments, and the samples were stable under the alternating temperature cycling.

For silicone rubber samples exposed to the simulated solution and air up to 100 cycles (Figure 5b), the intensity of the absorption bands between 1010 cm^−1^ and 1080 cm^−1^ decreased more sharply than those of the samples exposed to other environments. The intensity of the other absorption bands did not change obviously.

For silicone rubber samples exposed to the de-ionized water under the alternating temperature cycling from −20 °C to 90 °C up to 150 cycles (see Figure 5c), the intensity of the absorption bands had little change. For silicone rubber samples exposed to the acid solutions and air under the alternating temperature cycling from −20 °C to 90 °C up to 150 cycles (see Figure 5c), the intensity of the absorption bands between 1010 cm^−1^ and 1080 cm^−1^ decreased more sharply than those of the samples exposed to de-ionized water.

For silicone rubber samples exposed to the testing environments up to 200 cycles under the alternating temperature cycling from −20 °C to 90 °C (Figure 5d), the intensity of all the absorption bands had changes. For silicone rubber samples exposed to air up to 200 cycles (Figure 5d), the intensity of the absorption bands between 1010 cm^−1^ and 1080 cm^−1^, 793 cm^−1^, 864 cm^−1^ and 1260 cm^−1^ decreased dramatically.

From Figure 5, it can be seen that the intensity of all the absorption bands for samples in air under the alternating temperature cycling from −20 °C to 90 °C changed significantly, whereas they did not change apparently for samples exposed to other environments under the same conditions. The intensity of absorption bands of the samples in air for 200 cycles from −20 °C to 90 °C decreased more obviously. The decrease in intensity of absorption bands suggested breaking of Si–O–Si or decomposition of methyl groups attached to silicon atoms. The results show that alternating temperature cycling and environment had significant effect on the chemical degradation for the samples. Accordingly, ATR-FTIR data could be used as explanations for variation of weight and surface hardness.

**Figure 6:**
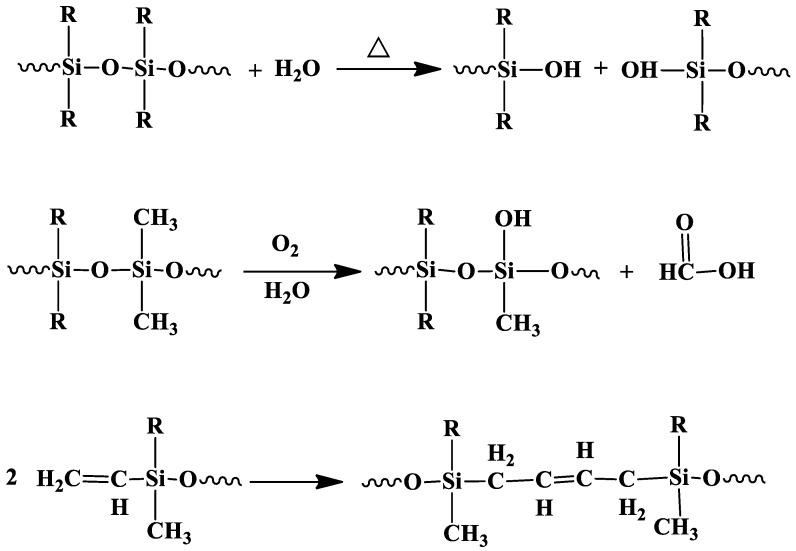
Hydrolysis, oxidation and cross-linking of Silicone rubbers.

From the ATR-FTIR results it can be concluded that there were significant chemical changes in silicone rubber backbone after the silicone rubber samples were exposed to the test environments. Both Si–CH_3_ and Si–O–Si signals decreased mostly significantly for the samples under temperatures cycling after 200 alternating temperature cycles in the air. It can be obtained that the chemical degradation is probably due to hydrolysis and chain cleavage of silicone rubbers. Si–O–Si in the silicone rubber backbone is stable, and the Si–O–Si could be converted to Si–O when it was exposed to the aging environment (Figure 6) [21,42].

## 4. Conclusions

Degradation of silicone rubber, a potential elastomeric gasket material used as sealing in fuel cells, was studied in a simulated PEMFC environment, weak acid solution, de-ionized water and air under temperature cycling from −20 °C to 90 °C. The conclusions can be drawn as follows:

(1) The aging environment and temperature cycles have an effect on the degradation of silicone rubbers as evidenced by weight loss. The weight loss increased with the concentration of the acidic solution and number of temperature cycles. In particular, the weight loss is more significant in solution than that in air.

(2) The silicone rubbers have a slight increase in hardness that indicates that the damage of the fillers in silicone rubbers in acid and neutral solution is more severe than that in air. SEM investigation indicates that surface topography of the rubbers exhibited cycle-dependent degradation, and the degradation started from surface holes and finally more and bigger void formation after exposure to the test environments.

(3) ATR-FTIR spectrometry results reveal that the surface chemistry changed significantly as an indication of the chemical degradation of the silicone rubber material exposed to the test environments. The degradation mechanisms of the silicone rubber could proceed via de-crosslinking through oxidation and hydrolysis of crosslink sites and chain cleavage in the backbone accompanied by the damage of the fillers in rubber.

The current work aims to show the chemical degradation and degradation mechanisms on the silicone rubber material. This study may provide guidance for improvement on durability of silicone rubbers as seals for PEMFC applications.

## Figures and Tables

**Table 1:** Geometric parameters of the Silicone rubber sample.

Area (mm^2^)	4000
Length (mm)	100
Width (mm)	40
Thickness (mm)	0.5

**Table 2:** Percent weight loss per cycle of the samples in different testing environments after temperature cycling.

Testing Cycle	Testing Environment (%)			
	pH = 3.35	pH = 5	pH = 7	AIR
50th	0.0060	0.0050	0.0050	0.0024
100th	0.0037	0.0033	0.0028	0.0014
150th	0.0031	0.0030	0.0020	0.0018
200th	0.0032	0.0025	0.0019	0.0017
